# Granite Dust and Silica Fume as a Combined Filler of Reactive Powder Concrete

**DOI:** 10.3390/ma17246025

**Published:** 2024-12-10

**Authors:** Andriy Huts, Janusz Konkol, Vitalii Marchuk

**Affiliations:** 1 Faculty of Civil and Environmental Engineering and Architecture, Rzeszow University of Technology, 35959 Rzeszow, Poland; janusz.konkol@prz.edu.pl; 2 Institute of Civil Engineering and Architecture, National University of Water and Environmental Engineering, 33028 Rivne, Ukraine; v.v.marchuk@nuwm.edu.ua

**Keywords:** reactive powder concrete, granite dust, silica fume, compressive strength, water absorption, mathematical experiment planning

## Abstract

By volume, cement concrete is one of the most widely used construction materials in the world. This requires a significant amount of Portland cement, and the cement industry, in turn, causes a significant amount of CO_2_ emissions. Therefore, the development of concrete with a reduced cement content is becoming an urgent problem for countries with a significant level of production and consumption of concrete. Therefore, the purpose of this article is to critically investigate the possibility of using inert granite dust in combination with highly active silica fume in reactive powder concrete. The main physical and mechanical properties, such as the compressive strength at different curing ages and the water absorption, were studied using mathematical planning of experiments. The consistency and microstructure of the reactive powder concrete modified with granite dust in combination with silica fume were also analyzed. Mathematical models of the main properties of this concrete are presented and analyzed, and the graphical dependencies of the influence of composition factors are constructed. A more significant factor that affects the compressive strength at all curing ages is the silica fume content, increases in which to 50 kg/m^3^ lead to a 25–40% increase in strength at 1 day of age, depending on the granite dust content. In turn, an increase in the amount of granite dust from 0 kg/m^3^ to 100 kg/m^3^ in the absence of silica is followed by an increase in strength of 8–10%. After 3 days of curing, the effect of granite dust becomes more significant. Increases in the 28-day strength of 25%, 46% and 56% were obtained at a content of 50 kg/m^3^ of silica fume and 0 kg/m^3^, 100 kg/m^3^ and 200 kg/m^3^ of granite dust in concrete, respectively. It is shown that the effect of inert granite dust is more significant in combination with silica fume at its maximum content in the range of variation. The pozzolanic reaction between highly active silica and Ca(OH)_2_ stimulates the formation of hydrate phases in the space between the grains and causes the microstructure of the cement matrix to compact. In this case, the granite dust particles act as crystallization centers.

## 1. Introduction

The problem of reducing carbon emissions has become a critical global priority, which has led to large-scale research and the development of various technological solutions aimed at reducing CO_2_ emissions. By volume, cement concrete is the most widely used construction material in the world. This requires a significant amount of Portland cement, and the cement industry is a major source of energy consumption and carbon emissions, accounting for approximately 7% of global CO_2_ emissions [1]. Currently, significant efforts are being made to reduce its environmental impact. This can be partially solved by managing quarry waste [2]. Over the past few decades, various methods have been proposed to reduce its environmental impact and use it in concrete production. In this sense, the use of secondary materials of anthropogenic origin, including granite dust in reactive powder concrete, can be considered as a modern approach to reducing resource consumption and CO_2_ emissions.

Reactive powder concrete (RPC), developed at the end of the twentieth century, is a new generation of concrete with a range of specific properties that allows for the creation of unique buildings and structures using advanced technologies. This type of concrete has increased density, strength, crack resistance, and improved deformation properties compared to those of conventional concrete [3,4,5]. RPC is a type of special concrete that does not contain coarse aggregates (size greater than 2 mm). Instead, chemically active fine powders (fly ash, silica fume, ground slag, metakaolin, etc.) are used. The fine components of this type of concrete are chemically transformed during curing, which is described by the term ‘reactive powder’.

Compared to conventional fine-grained concrete, RPC has higher mechanical characteristics. This can be explained by the fact that, by eliminating the coarse aggregate fraction, the homogeneity of the concrete microstructure is increased. Additionally, by optimizing the grain composition of the concrete mixture in combination with the addition of active mineral additives, the content of the cement matrix increases and the density of the concrete increases [6,7,8]. The use of RPC in construction has been confirmed by studies on its effectiveness [9].

The addition of active mineral additives to cement matrix materials improves their mechanical properties and durability [10]. In such composites, these additives can act as pozzolanic, cementitious, or filling materials [11].

Inert materials, such as granite dust [12,13] or basalt dust [14], cause the ‘microfiller effect’ to act as crystallization centers, i.e., fill the spaces in the microstructure of the cement paste and physically stimulate cement hydration in the first days of curing, thus compacting the structure of the cement matrix [15,16]. These materials act as nucleation points and growth points for C–S–H and other hydrates [17]. According to [18], several researchers consider that finely dispersed SiO_2_ particles up to 5 μm in size may have pozzolanic activity. Berodier and Scrivener [16] found that replacing 20–70% of the cement with quartz with diameters of 4, 13 and 18 microns physically stimulates the hydration of the cement clinker in the initial stage of curing (in the first hours). Kadri et al. [19] noted that 10% of the cement replaced with quartzite (~75% quartz) with diameters of 2.6, 5.5 and 11 μm did not lead to a significant change in the strength.

Studies by Snellings, Mertens and Elsen [20] found that the use of a wide range of natural and technogenic cementitious materials for RPC allows for a directed effect on the processes of structure formation and hydrate phases in the non-clinker part with the formation of a fine crystalline structure, which increases the strength of the cement matrix due to its compaction. Reducing the heat of hydration of cement is the reason for the increase in the content of active mineral additives. This, in turn, also reduces shrinkage deformations [21,22].

The combination of inert granite dust and pozzolanic additives can eliminate some of the disadvantages of using only one component, namely, accelerating the pozzolanic reaction during cement hydration and compacting the structure of the cement matrix, since these materials are characterized by opposite properties [23].

Silica fume is a highly dispersed active cementitious material. It reacts chemically with calcium hydroxide, which is formed during the hydration of cement. This, in turn, leads to the formation of additional amounts of (C–S–H) and other hydrated compounds as a result of the pozzolanic reaction. Silica fume also fills the spaces between Portland cement particles, acting as a microfiller [24].

The aim of this work is to improve the physical and mechanical properties (compressive strength, water absorption, etc.) of reactive powder concrete by partially replacing the cement with a complex filler consisting of a mixture of inert granite dust and highly active fine silica fume.

Thus, the use of granite dust in combination with active mineral additives as fillers can reduce the energy consumption and CO_2_ emissions while producing concrete and mortars by reducing the amount of Portland cement or improving their physical and mechanical properties without increasing the content of cement.

The novelty of this work is to prove the feasibility of using inert granite dust in combination with highly active silica fume in the composition of reactive powder concrete and to study its main strength properties using mathematical experiment planning methods.

## 2. Materials and Methods

### 2.1. Materials Used

The materials used in the research were as follows.

- Portland cement CEM I 42.5 N, produced by “Ozarow S.A.”, Ozarow, Poland. The physical and mechanical properties of the cement comply with the requirements of PN-EN 197–1:2012 [25]. Mineral composition of cement clinker: C3S—64.7%, C2S—12.5%, C3A—9.5%, C4AF—6.8%;
- Quartz sand of 0–2 mm fraction, produced by “Kruszgeo S.A.”, Mrowla, Poland, according to PN-EN 12620+A1:2010 [26];
- Superplasticizer (SP) MasterGlenium ACE 560—highly effective liquefaction admixture based on polycarboxylate ether technology, produced by “Master Builders Solution”, Myslenice, Poland, according to PN-EN 934–2+A1:2012 [27];
- Granite dust (GD)—waste material obtained during the process of making crushed granite stone (aggregate) at the “Strzegom” quarry, Strzegom, Poland. Physical properties of granite dust are shown in Table 1;
- Silica fume (SF) for concrete category 1, SILIMIC—undensified, produced by “Re Alloys Poland”, Laziska Gorne, Poland, according to EN 13263–1:2005 [28]. The chemical and physical parameters of the silica fume are shown in Table 2.

**Figure 1:**
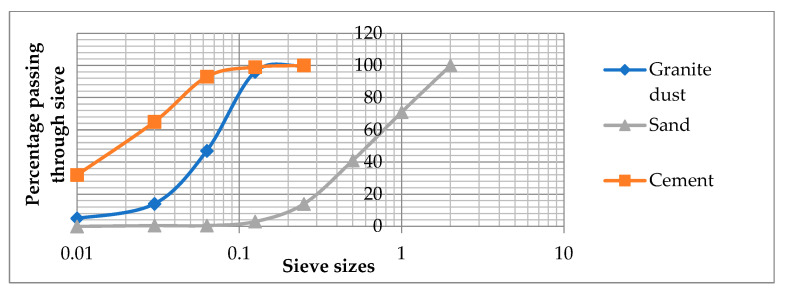
Granite dust, sand and cement particle size distribution.

The fractional composition of cement, sand and granite dust is shown in Figure 1.

### 2.2. Research Methods

This article presents a study of the basic physical and mechanical properties of concrete mixtures and reactive powder concrete modified with granite dust and silica fume using methods that comply with the requirements of current standards. Physicochemical and physicomechanical research methods were used.

**Figure 2:**
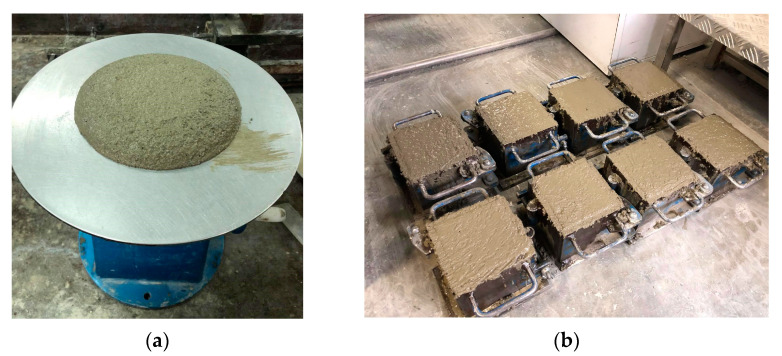
Fresh reactive concrete mixture before (**a**) and after molding (**b**).

The consistency of fresh concrete mixtures was investigated using the cone slump method in accordance with the requirements of PN-EN 12350–2:2019 [29]. A fresh mix of reactive powder concrete is shown in Figure 2a. Figure 2b shows the process of forming fresh concrete into a mold.

**Figure 3:**
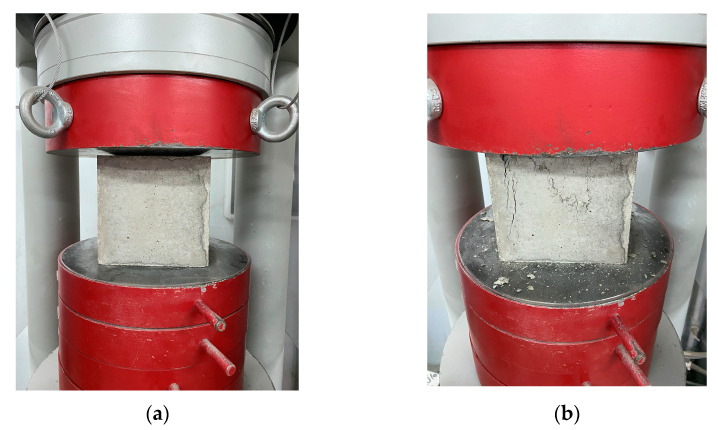
Compressive strength of the RPC: (**a**) sample before the test; (**b**) sample after the test.

The compressive strength at the appropriate curing age was determined in cubic samples according to PN-EN 206+A1:2016 [30]. Compressive strength tests were performed on a FormTest PRUFSYSTEME ALPHA 3–3000 S tester, manufactured by “Form + Test Seidner & Co. GmbH”, Riedlingen, Germany. To evaluate the effect of granite dust and silica fume on concrete strength properties, standard cubic specimens (15 × 15 × 15 cm) were prepared and cured under normal conditions (humidity 95 ± 5%, temperature 20 ± 2 °C). The compressive strength of 6 specimens was determined at the age of 1, 3 and 28 days. The compressive strength of the sample before the test is shown in Figure 3a and the sample after the test is shown in Figure 3b.

**Figure 4:**
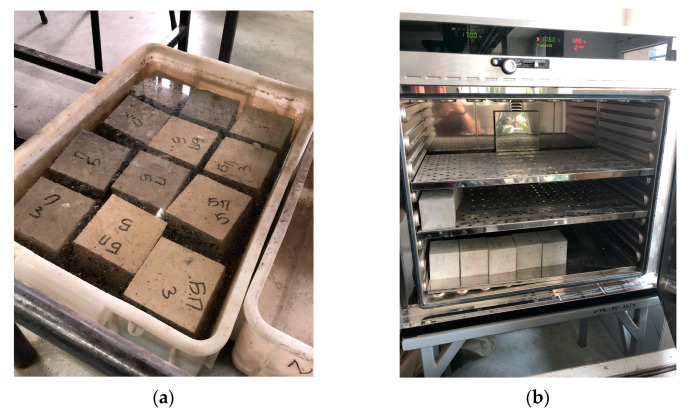
Water absorption test of RPC: (**a**) impregnation of the samples; (**b**) drying of the samples in a laboratory drying chamber.

Water absorption was determined according to PN-EN 206+A1:2016 [30] by measuring the change in weight of the samples before and after drying in a laboratory climate chamber. Figure 4a shows the impregnation of the water in the samples and Figure 4b shows the drying process.

Microstructure analysis was performed on concrete samples that were mechanically separated using the scanning electron microscopy method. For this study, a JSM–5500 LV microscope (manufactured by “JEOL”, Tokyo, Japan) was used.

The results of the experimental studies presented in the article were processed using statistical methods of mathematical modeling. Conducting experimental studies with the widespread use of mathematical experiment planning allows the process of laboratory experiments to fully algorithmize according to a scheme that is optimal in terms of the amount of experimental work and meets statistical requirements [31]. A statistical analysis of the results obtained allows one to obtain mathematical models in the form of a second-order regression equation. These equations reflect the direct relationship between the input and output parameters controlled by quantitative factors of influence. Mathematical models can also be used to analyze the entire process under study, perform technological calculations, and perform certain optimizations. In addition, the statistical analysis was supplemented by calculating the coefficient of determination for the regression models obtained, analyzing the significance of the equation coefficients and the adequacy of the regression model.

The task of mathematical modeling is to obtain an idea of the response surface of the factors (graphical dependence), which can be analytically represented as a function, as illustrated in Equation (1):

*M_{y}_ = φ(X*_1_,*X*_2_,*X*_3_,…,*X_n_*), (1)

where *y* is the optimization parameter, that is, the output parameter of the system; *X_i_* represents the variable factors of the same system.

The most convenient method is to represent the unknown response function as a polynomial, which is shown in the form of Equation (2):

(2) $$Y=\beta_{0}+\sum_{i=1}^{n}\beta_{i}X_{i}+\sum_{i=1}^{n}\beta_{ii}X_{i}^{2}+\sum_{i\neq j}\beta_{ij}X_{i}X_{j}+\ldots$$

where *X_i_* and *X_j_* are independent factors that can be varied during the experiments; *β*_0_, *β_i_*, *β_ij_* and *β_ii_* are theoretical regression coefficients.

The most important purpose of mathematical modeling is to optimize the characteristics of a technological process. Mathematical models can be used to solve technological problems that differ, as follows:

- The number of equations included in the system of inequalities (constraints);
- The nature of the functions;
- The number of factors;
- The nature of dependencies between factors;
- The degree of intensity and nature of changes in the factors over time.

### 2.3. Concrete Mixture Proportion

In determining the main properties of reactive powder concrete filled with granite dust in combination with silica fume, a series of experiments were carried out according to the algorithm of a two-factor experimental plan of the second order of type B2, close to the optimum criterion of D. Obtaining the D-optimal plan is based on the optimal positioning of the experimental points in the simplex, within the range of variation in the factors [31]. The conditions for planning the experiment are given in Table 3. The variable factors were as follows: *X*_1_—granite dust content, which varied from 0 to 200 kg/m^3^, and *X*_2_—silica fume content, from 0 to 50 kg/m^3^. The water consumption in all mixtures was constant at 205 L/m^3^. Additionally, a polycarboxylate superplasticizer was added to the RPC at an amount of 0.7% by weight of Portland cement.

## 3. Results and Discussion

The composition of the concrete mixtures and the experiment planning matrix are shown in Table 4. The obtained results are shown in Table 5. The results are supplemented by the standard error of the mean value.

An analysis of variance of the effect of the input quantities on the output quantities using the F–Snedecor test showed a highly statistically significant effect of the input quantities (variables in the test plan) on the output quantities (properties tested). For both the compressive strength after 1, 3 and 28 days of curing and the water absorption, borderline significance levels close to zero were obtained.

In the case of the compressive strength after 1, 3 and 28 days of curing and water absorption, the variance homogeneity test was performed using the Brown–Forsythe test. The limit significance levels obtained are 0.79, 0.84 and 0.20 for the compressive strength after 1, 3 and 28 days, respectively, indicating the homogeneity of the variance of the different concrete series.

Using a statistical analysis of the experimental data obtained, quadratic mathematical models of strength (*f*^1^*_c,cube_*, *f*^3^*_c,cube_*, and *f*^28^*_c,cube_*) and water absorption (*W*) were constructed in the form of polynomial regression equations of coded variables (Equations (3)–(6)). The corresponding Fisher criteria confirm the adequacy of the models obtained. In addition, the values of the coefficients of determination are given along with the model. The value of this coefficient is, at the same time, information on to what extent the variation in a given parameter (compressive strength or water absorption) is explained by the variation in the proportion of a given additive and to what extent by other factors not included in the study or random factors.

Compressive strength at 1 day of age:

*f*^1^*_c,cube_ =* 24.7 + 1.8 × *X*_1_ + 3.3 × *X*_2_ + 1.0 × *X*_1_^2^ − 0.5 × *X*_2_^2^ − 0.4 × *X*_1_ × *X*_2_, *R*^2^ = 0.87 (3)

Compressive strength at the age of 3 days:

*f*^3^*_c,cube_ =* 45.8 + 3.7 × *X*_1_ + 3.2 × *X*_2_ + 0.9 × *X*_1_^2^ + 0.1 × *X*_2_^2^ + 0.9 × *X*_1_ × *X*_2_, *R*^2^ = 0.71 (4)

Compressive strength at the age of 28 days:

*f*^28^*_c,cube_ =* 77.3 + 7.4 × *X*_1_ + 10.2 × *X*_2_ − 3.2 × *X*_1_^2^ − 1.6 × *X*_2_^2^ + 2.1 × *X*_1_ × *X*_2_, *R*^2^ = 0.84 (5)

Water absorption at the age of 28 days:

*W* = 4.21 − 0.60 × *X*_1_ − 1.08 × *X*_2_ − 0.13 × *X*_1_^2^ + 0.12 × *X*_2_^2^ + 0.15 × *X*_1_ × *X*_2_, *R*^2^ = 0.95 (6)

Dependencies (Equation (7)) were used to convert the values of the parameters studied in the composition of concrete mixtures into a coded form:

(7) $$X_{1}=\frac{GD-100}{100};\;X_{2}=\frac{SF-25}{25}$$

### 3.1. Consistency

**Figure 5:**
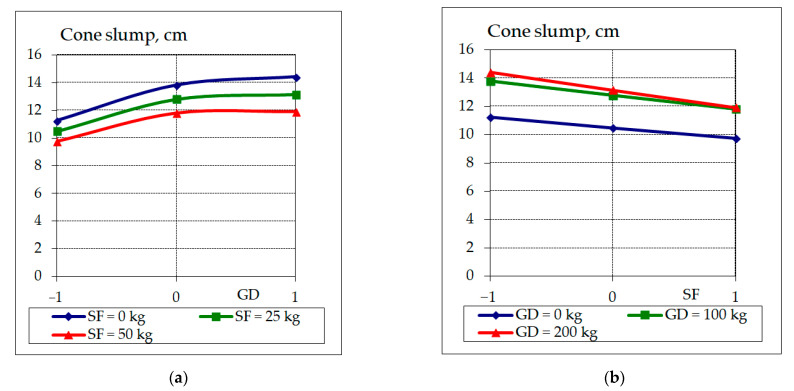
Graphical dependencies of the consistency of RPC, where the content of GD −1 = 0 kg; 0 = 100 kg; +1 = 200 kg (**a**); the content of SF −1 = 0 kg; 0 = 25 kg; +1 = 50 kg (**b**).

The results of the consistency study are shown in Figure 5.

Consistency is one of the main parameters that affect the workability of a concrete mixture. The analysis of the results in Table 5 and Figure 5 shows that with a constant binder content (CEM + SF) and constant water consumption, the content of granite dust is decisive and an increase in its content increases the workability of the concrete mixture (Figure 5a). The addition of silica fume leads to a decrease in workability (Figure 5b), which is explained by the fact that the water demand for the highly dispersed silica fume is higher than the water demand for cement. The dependence of the workability of the concrete mixture on the content of a complex additive containing granite dust was found to be extreme and its optimal content should not exceed 30–40% of the weight of the cement, as confirmed by studies [12,13]. Dispersed active mineral additives partially neutralize the plasticizing effect of the superplasticizer [32], which may be the reason for the thickness of some of the concrete mixtures with the highest content of granite dust and silica fume within the range of variation. The dosage and nature of superplasticizers play an important role in ensuring the required workability of concrete mixtures with the addition of dispersed additives (GD, SF, etc.).

### 3.2. Compressive Strength

**Figure 6:**
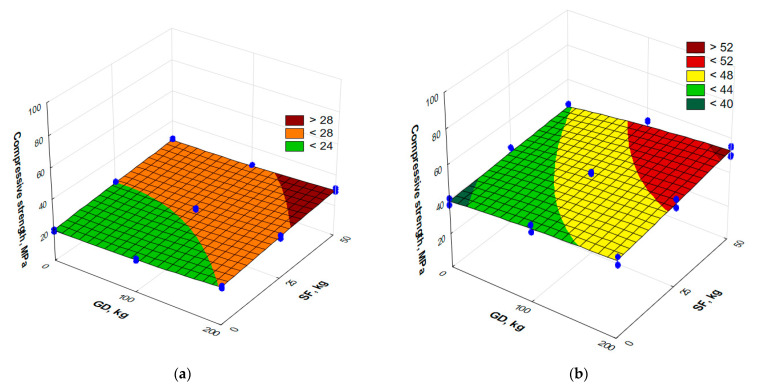
Response surface of compressive strength at the age of 1 day (**a**) and 3 days (**b**) of reactive powder concrete modified with granite dust and silica fume.

The compressive strength of the RPC at the age of 1 and 3 days is shown in Figure 6.

By analyzing the experimental–statistical model of the compressive strength obtained at the age of 1 day (Equation (3)), it can be concluded that an increase in the content of silica fume (*X*_2_) to 50 kg/m^3^ is a more significant factor of influence. This leads to an increase in the compressive strength of 25–40%, depending on the content of granite dust. The impact of this factor is almost linear (Figure 6a). An increase in the amount of granite dust (*X*_1_) from 0 kg/m^3^ to 100 kg/m^3^ in the absence of silica fume is followed by an increase in strength at 1 day of 8–10%. At the points with an SF and medium GD consumption of up to 100 kg/m^3^, the positive effect on the initial strength of the granite filler is less significant. An increase in the amount of GD added to 200 kg/m^3^ results in a 10–15% increase in strength with a change in the content of SF within the range of variation. According to the experimental–statistical model of the compressive strength at the age of 3 days (Equation (4) and Figure 6b), it can be observed that the effect of GD becomes more significant, and the addition of SF allows higher strength values to be obtained with an increase in the content of GD within the range of variation.

**Figure 7:**
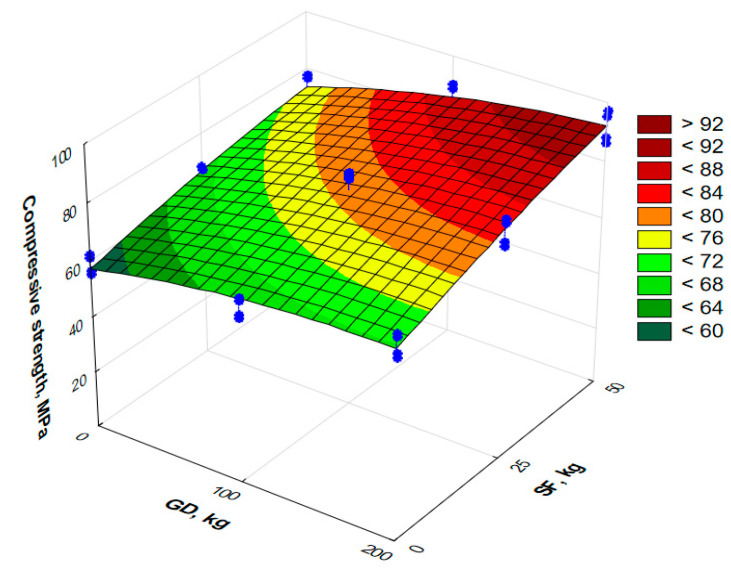
The surface of response to compressive strength at the age of 28 days of reactive powder concrete modified with granite dust and silica fume.

The compressive strength of the RPC at the age of 28 days is shown in Figure 7.

The analysis of the experimental–statistical model obtained for the compressive strength at 28 days (Equation (5) and Figure 7) allows one to note that the content of SF is also a more significant factor: an increase of 50 kg/m^3^ leads to an almost linear increase in the strength of 25%, 46% and 56% when the content of granite filler in the concrete is 0 kg/m^3^, 100 kg/m^3^ and 200 kg/m^3^, respectively. It is necessary to note the significant effect of the interaction of factors. Obviously, achieving high concrete strength is possible with the appropriate optimization of the mixtures. Table 6 compares the percentage increase in the compressive strength of all the RPC mixtures studied. Mixture No. 4 was selected as the point of reference (the content of granite dust and silica fume is 0 kg/m^3^).

At the maximum consumption of silica fume, the effect of granite dust is more significant. This can be explained by the fact that the microstructure of the cement matrix is compacted due to the partial replacement of sand with granite dust, since the last has a much smaller grain size. This may be the main reason for the increase in the concrete strength with the addition of granite dust (Figure 6 and Figure 7), which is confirmed by references [12,13,14].

At the same time, the partial replacement of Portland cement with a complex additive containing GD and SF allows for an increase in the compressive strength at all the curing periods, and first of all due to the much higher pozzolanic activity of the SF. The binding of Ca(OH)_2_ to amorphous SiO_2_ begins at an early age when adsorbed on SF particles. This produces low-base calcium hydrosilicates. The change in balance between C–S–H (I) and C–S–H (II) in the composition of cement stone with silica fume depends on the dose of SF and the amount of SiO_2_ in the inert filler. With an increase in the amount of SF, the content of C–S–H (I) increases and that of C–S–H (II) decreases, which is in accordance with the studies in references [16,32].

The effectiveness of filling the cement system with fine GD particles containing SF in combination with a superplasticizer can be explained by the fact that due to interrelated colloidal chemical and physical processes based on the gradual flow of a moderate amount of superplasticizer into the liquid phase of the cement system containing mineral additives, it is possible to control the rheological properties of concrete mixtures and modify the structure of cement stone. Each particle of the modifier is an aggregate consisting of ultrafine particles of SF or its mixture with larger particles of GD. The dispersed materials are evenly coated with a thin layer of a superplasticizer additive that “glues” the particles of mineral components together, leading to the formation of strong and stable bonds in the air environment.

### 3.3. Microstructure

**Figure 8:**
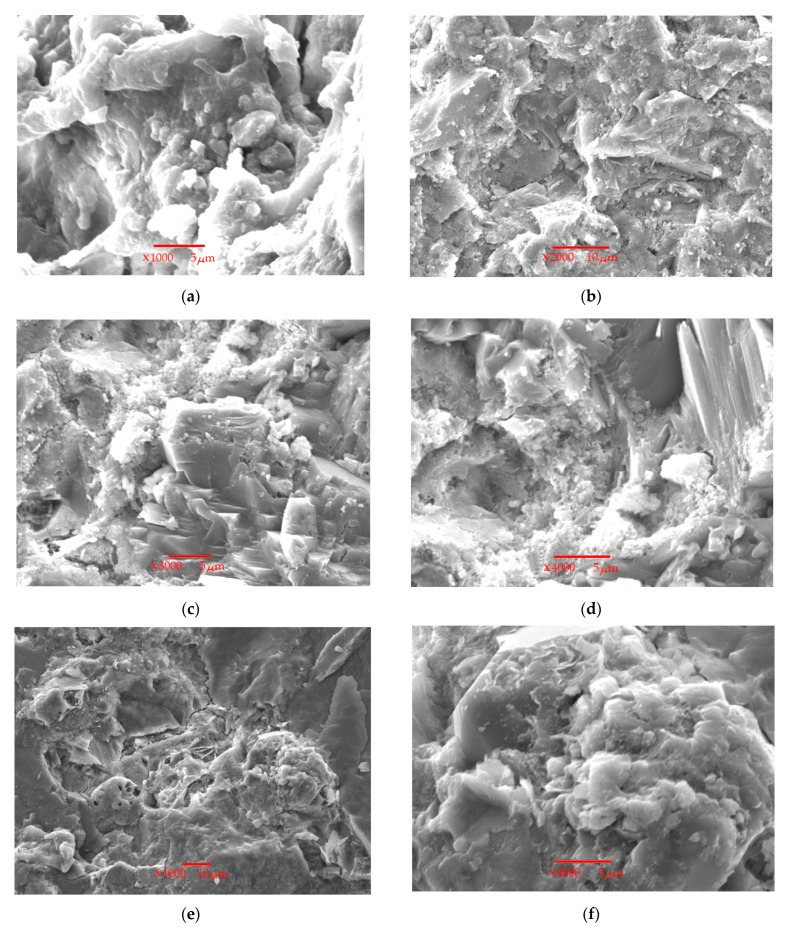
Microstructure of reactive powder concrete at the age of 28 days: (**a**) control sample; (**b**) GD = 100 kg/m^3^; (**c**) GD = 200 kg/m^3^; (**d**) GD = 100 kg/m^3^ + SF = 25 kg/m^3^; (**e**,**f**) GD = 200 kg/m^3^ + SF = 50 kg/m^3^.

The microstructure of the RPC at the age of 28 days is shown in Figure 8.

As a result of the hydration of Portland cement, the presence of calcium hydrosilicate (C–S–H) surrounding the fine aggregate particles was detected in the samples. Other hydrated compounds are also visible in the form of lamellar particles of calcium hydroxide and ettringite. Portlandite was detected only in the control sample (Figure 8a) and in the composition with GD (Figure 8b,c). This can be explained by the fact that the pozzolanic activity of silica fume at the age of 28 days contributes to an increase in the density of the C–S–H phase, which in turn densifies the microstructure of the cement composite. The photo of the microstructure of the samples with the addition of GD, in turn, is very similar to that of the control sample, which indicates its low reactivity. However, at the same time, the hydration products settle in small particles of granite dust, which leads to the densification of the structure. These particles also form the so-called ‘crystallization centers’. Furthermore, a C–S–H phase was found on the surface of the calcium hydroxide crystals. This may indicate a higher concentration of calcium hydroxide and a higher C–S–H content with a higher Ca/Si ratio in the microstructure. Figure 8d–f show that the pozzolanic activity densifies the microstructure and makes it more compact, but there are no Ca(OH)_2_ crystals. This can be explained by the high absorption of Ca(OH)_2_ by silica fume. It should also be noted that the interface between the granite dust and Portland cement can be compacted as a result of the pozzolanic reaction through the secondary formation of C–S–H. This may show that due to the pozzolanic activity, there is an improvement in mechanical properties, a decrease in water absorption, and an increase in the strength of concrete based on a composite additive containing silica fume in combination with granite dust.

### 3.4. Water Absorption

**Figure 9:**
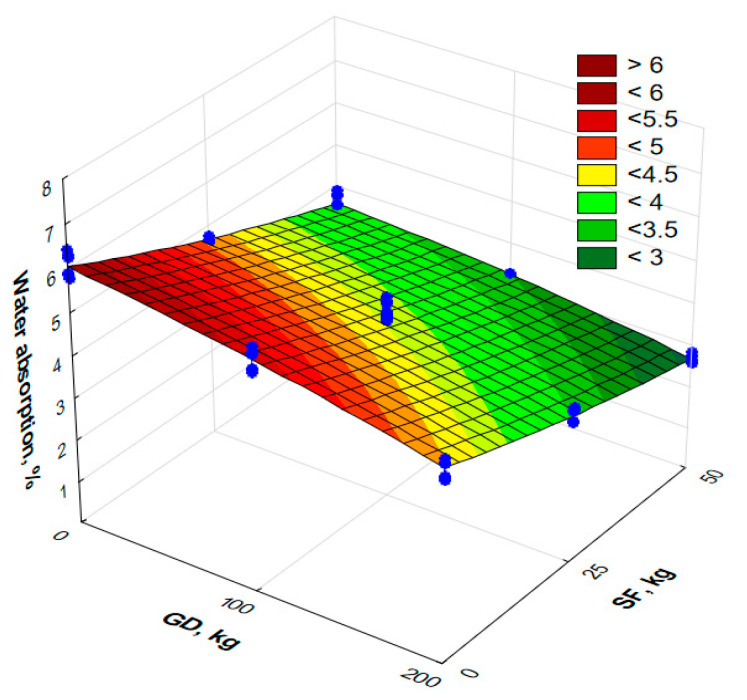
Response surface to water absorption of reactive powder concrete modified by granite dust and silica fume at the age of 28 days.

Based on the results obtained (Table 5), a response surface was constructed that shows the effect of the type and amount of mineral additives on the water absorption parameters (Figure 9).

The addition of granite dust significantly affects the water absorption of concrete, as shown in Equation (6). When the sand is replaced with granite dust, a clear tendency is observed to decrease water absorption (Figure 9). The combination of 100 kg/m^3^ and 200 kg/m^3^ of GD with 25 kg/m^3^ and 50 kg/m^3^ of SF leads to a decrease in water absorption from 4.7% and 3.6% to 3.5% and 2.7%, respectively. Water absorption can be reduced as a result of the pozzolanic reaction of SF in the presence of GD as crystallization centers. This also leads to the compaction of the microstructure of the cement matrix. The results of the study indicate that with an increase in the amount of a complex additive containing finely dispersed GD and SF, the specific surface of the concrete components increases, which is accompanied by a decrease in water absorption, as confirmed in [33].

## 4. Conclusions

Based on the results of the reactive powder concrete tests with the addition of granite dust and silica fume, the following conclusions can be drawn.

- The addition of granite dust to reactive powder concrete makes it possible to obtain mixtures with increased workability by 2–3 cm;
- The binding of Ca(OH)_2_ to amorphous SiO_2_ begins at an early age during adsorption on silica fume particles;
- Analyzing the microstructure of the cement stone, it can be observed that small particles of granite dust act as crystallization centers. This can help accelerate the initial phase of chemical curing;
- An increase in the amount of silica fume up to 50 kg/m^3^ leads to an increase in strength at the age of 1 day from 19.8 MPa to 27.1 to 30.1 MPa, depending on the content of granite dust;
- The addition of granite dust at an amount of 100–200 kg/m^3^ to the reactive powder concrete mixture in combination with silica fume at an amount of 25–50 kg/m^3^ leads to improved mechanical parameters and the increased compressive strength of the concrete at different curing times;
- The 28-day compressive strength of 73.5 MPa, 85.6 MPa and 92.0 MPa was obtained at a content of 50 kg/m^3^ of silica fume and 0 kg/m^3^, 100 kg/m^3^ and 200 kg/m^3^ of granite dust in the concrete mixture, respectively.
- The addition of a dispersed granite filler in combination with active pozzolanic silica fume ensures low water absorption.
- A significant effect of the interaction of factors is observed and it is obvious that achieving a high concrete strength is possible with the appropriate optimization of the composition of the mixture.

The possibility of using technogenic granite dust in combination with silica fume as aggregates for reactive powder concrete will reduce the energy consumption and CO_2_ emissions in concrete production.

Studies have shown that a more significant effect of granite dust on the analyzed parameters is observed in combination with silica fume at its maximum level in the range of variation. It should be noted that a positive effect is observed with a consumption of granite dust of 100 kg/m^3^ and silica fume of 25 kg/m^3^. In terms of the results obtained (compressive strength, water absorption and microstructure density), the best RPC mixture is the one with the maximum consumption of granite dust and silica fume, i.e., 200 kg/m^3^ of GD content and 50 kg/m^3^ of SF content. In this case, compared to concrete without GD and SF, the compressive strength increases by 52%, 35% and 56% at the age of 1 day, 3 days and 28 days, respectively, and water absorption decreases from 6.1% to 2.7%. The pozzolanic reaction between highly active silica fume and Ca(OH)_2_ stimulates the formation of hydrate phases in the space between the grains and causes the microstructure of the cement matrix to compact. In this case, granite dust particles play the role of crystallization centers.

## Figures and Tables

**Table 1:** Physical parameters of granite dust.

No.	Specification	Value
1	Bulk density, kg/m^3^	910
2	Specific surface, m^2^/kg	250
3	Humidity, %	0.4
4	Clay content, %	0.3
5	Water-soluble contaminants, %	none

**Table 2:** Chemical and physical properties of silica fume.

No.	Specification	Value
1	SiO_2_ content, %	Min. 85
2	Si content, %	Max. 0.4
3	CaO content, %	Max. 1.0
4	SO_3_ content, %	Max. 2.0
5	Na_2_O equivalent, %	Max. 3.0
6	Cl^−^ content, %	Max. 0.3
7	Specific surface, m^2^/g	Min. 15
8	Activity index	Meets
9	Bulk density, kg/m^3^	350

**Table 3:** Experiment planning conditions.

No.	Factors		Level of Variation			Range
	Coded Form	Origin Form	−1	0	+1	
1	*X*_1_	Granite dust consumption (GD), kg/m^3^	0	100	200	100
2	*X*_2_	Silica fume consumption (SF), kg/m^3^	0	25	50	25

**Table 4:** Composition of concrete mixtures and experiment planning matrix.

No. Point of Matrix	Coded Values of Factors		Concrete Mixture, kg/m^3^				
	*X*_1_	*X*_2_	GD	CEM	SF	Sand	SP
1	+1	+1	200	500	50	1427	3.5
2	+1	−1	200	550	0	1431	3.9
3	−1	+1	0	500	50	1628	3.5
4	−1	−1	0	550	0	1627	3.9
5	+1	0	200	525	25	1431	3.7
6	−1	0	0	525	25	1628	3.7
7	0	+1	100	500	50	1530	3.5
8	0	−1	100	550	0	1529	3.9
9	0	0	100	525	25	1530	3.7
10	0	0	100	525	25	1530	3.7
11	0	0	100	525	25	1530	3.7

**Table 5:** Experimental results obtained.

No. Point of Matrix	Coded Values of Factors		Cone Slump, cm	Compressive Strength, MPa, at the Age			Water Absorption, % W
	*X*_1_	*X*_2_		1 Day	3 Days	28 Days	
1	+1	+1	11.5	30.1 ± 0.77	52.5 ± 1.55	92.0 ± 2.71	2.7 ± 0.04
2	+1	−1	14.5	24.2 ± 0.52	44.7 ± 1.32	68.8 ± 2.02	4.5 ± 0.08
3	−1	+1	9.5	27.1 ± 0.60	43.1 ± 1.27	73.5 ± 2.16	3.7 ± 0.07
4	−1	−1	11.5	19.8 ± 0.52	38.8 ± 1.14	58.8 ± 1.73	6.1 ± 0.11
5	+1	0	13.5	27.3 ± 0.60	48.8 ± 1.44	80.3 ± 2.36	3.5 ± 0.07
6	−1	0	10.5	24.1 ± 0.56	42.0 ± 1.24	64.5 ± 1.90	4.5 ± 0.08
7	0	+1	12.5	27.4 ± 0.66	50.1 ± 1.47	85.6 ± 2.52	3.1 ± 0.06
8	0	−1	13.5	21.1 ± 0.53	42.8 ± 1.26	62.4 ± 1.82	5.4 ± 0.10
9	0	0	12.5	25.1 ± 0.60	46.0 ± 1.36	78.2 ± 2.30	4.2 ± 0.08
10	0	0	12.5	24.8 ± 0.73	45.3 ± 1.33	78.1 ± 2.30	4.1 ± 0.08
11	0	0	13.0	24.3 ± 0.44	45.1 ± 1.33	78.8 ± 2.31	4.5 ± 0.08

**Table 6:** Percentages of changes in compressive strength at different curing ages.

No. Point of Matrix	Granite Dust, Cement and Silica Fume Content, kg/m^3^			Percentage Change in Compressive Strength at Different Ages		
	GD	CEM	SF	1 Day	3 Days	28 Days
1	200	500	50	+52%	+35%	+56%
2	200	550	0	+22%	+15%	+17%
3	0	500	50	+37%	+11%	+25%
4	0	550	0	0% (19.8 MPa)	0% (38.8 MPa)	0% (58.8 MPa)
5	200	525	25	+38%	+26%	+37%
6	0	525	25	+22%	+8%	+10%
7	100	500	50	+38%	+29%	+46%
8	100	550	0	+7%	+10%	+6%
9	100	525	25	+27%	+19%	+33%
10	100	525	25	+25%	+17%	+33%
11	100	525	25	+23%	+16%	+43%

## Data Availability

The original contributions presented in this study are included in the article. Further inquiries can be directed to the corresponding author.
